# Just Waked up

**Published:** 1872-07

**Authors:** 


JUST WAKED UP.—Several years ago Dr. Sailsbury, of Cleveland, Ohio, published articles claiming to have discovered, by aid of the microscope, certain "algoid vegetations," which were to be found in the blood of persons having syphilis.* Little attention was paid to his assertions, and the matter was almost forgotten. But now, the foreign medical journals are wild over the discovery of the same "vegetations" by Dr. J. R. Chadwick, of Vienna, who claims to be the first discoverer of the phenomena. Of course, some attention will now be paid to Dr. Sailsbury, who will at once become a lion by reason of our foreign friends just waking up.

